# Dynamic modulation of the processing of unpredicted technical errors by the posterior cingulate and the default mode network

**DOI:** 10.1038/s41598-024-64409-6

**Published:** 2024-06-12

**Authors:** Zhiyan Wang, Markus Becker, Gregor Kondla, Henner Gimpel, Anton L. Beer, Mark W. Greenlee

**Affiliations:** 1 Faculty of Human Sciences, University of Regensburg, Universitätsstraße 31, 93053 Regensburg, Germany; 2 Faculty of Business, Economics and Social Sciences, University of Hohenheim, Schloss Hohenheim 1B, 70599 Stuttgart, Germany; 3 University of Regensburg, Sedanstraße 1, 93055 Regensburg, Germany

**Keywords:** Cognitive control, Problem solving, Human behaviour

## Abstract

The pervasive use of information technologies (IT) has tremendously benefited our daily lives. However, unpredicted technical breakdowns and errors can lead to the experience of stress, which has been termed technostress. It remains poorly understood how people dynamically respond to unpredicted system runtime errors occurring while interacting with the IT systems on a behavioral and neuronal level. To elucidate the mechanisms underlying such processes, we conducted a functional magnetic resonance imaging (fMRI) study in which 15 young adults solved arithmetic problems of three difficulty levels (easy, medium and hard) while two types of system runtime errors (problem errors and feedback errors) occurred in an unexpected manner. The problem error condition consisted of apparently defective displays of the arithmetic problem and the feedback error condition involved erroneous feedback. We found that the problem errors positively influenced participants’ problem-solving performance at the high difficulty level (i.e., hard tasks) at the initial stage of the session, while feedback errors disturbed their performance. These dynamic behavioral changes are mainly associated with brain activation changes in the posterior cingulate and the default mode network, including the posterior cingulate cortex, the mPFC, the retrosplenial cortex and the parahippocampal gyrus. Our study illustrates the regulatory role of the posterior cingulate in coping with unpredicted errors as well as with dynamic changes in the environment.

## Introduction

As new developments in information technologies (IT) increasingly pervade people’s daily lives, both benefits and challenges arise from our routine interactions with IT products. Technostress has emerged and been studied in recent years as a phenomenon to describe such challenges. Technostress is defined as an IT user’s experience of stress when interacting with technologies^1,2^. Such experiences can induce negative consequences on both behavioral and physiological responses^2–11^. Technostress can be separated into different sub-types, depending on the factors (techno-stressors) and sub-processes (challenge/hindrance/threat stress or eustress/distress) that induce the stressful experiences^1,8,12–14^.

One of the less studied but likely most encountered hindrance techno-stressors is techno-unreliability^4,13,15,16^. It refers to the lack of dependability and consistency of an IT system^4^. Techno-unreliability describes the phenomena when IT users “face system malfunctions and other IT hassles”^13^^, p.1462^. Common issues of unreliability are runtime errors (referred to as spontaneous runtime errors that occur during the execution of the computer codes), quality problems, or IT system breakdowns^4,17^. Techno-unreliability has been reported to lead to frustration and strain^18^ and hinder task performance^13,17^. Previous studies have used functional magnetic resonance imaging (fMRI) to describe participants’ brain responses to repetitively occurring and interruptive security warning messages^19–21^. Participants showed decreased neural responses to warning messages in the ventral visual pathways and the visual attention networks (inferior temporal gyrus, the inferior frontal gyrus and the dorsal medial prefrontal cortex) over repeated presentation of these messages^19,20^. They also showed increased neural responses in the medial prefrontal cortex and the retrosplenial cortex as part of the habituation process^19^. Moreover, Vance and colleagues^21^ have shown that the neural responses in the medial temporal lobe to disregarded warning messages can be modulated by the task difficulty levels.

While these studies focused on participants’ neural and physiological responses related to the presentation and habituation of error messages, it is largely unknown how such errors or system breakdowns dynamically influence people’s decision-making process to regulate their responses to achieve better behavioral performance. Furthermore, there is also a lack of systematic understanding of the underlying neural mechanisms associated with these dynamic processes while people attempt to cope with the unpredicted IT system breakdowns.

Two networks^22,23^ have been identified that are thought to play a role in the modulation of the decision-making process under uncertainty and stress: the executive network including the dorsolateral prefrontal cortex (DLPFC) and the basal ganglia^24–29^ and the default mode network centered around the posterior cingulate cortex (PCC) involving the retrosplenial cortex (RSC), the parahippocampal gyrus (PHG) and the medial prefrontal cortex (mPFC)^30–36^. Previous studies have proposed that the default mode network in particular the dorsal posterior cingulate cortex (dPCC) might play a significant role in representing different contexts and constraints for the decision-making environment^37–40^. The dPCC has also been found to be associated with environment or context uncertainty^41,42^ as well as the allocation of attention under uncertainty^43^. On the other hand, the executive control network might play a more important role in modulating each individual decision under a certain context^40^. While both networks can modulate how people respond to changes in the environment and dynamically adjust their strategies it is unclear which network plays a major role in the modulation of coping with unpredicted errors while interacting with IT products. Specifically, it is unclear whether responses to unpredicted errors were modulated based on individual situations (executive control network dominant) or based on the context in which the errors occurred (default mode network dominant).

Therefore, to address how technostress responses evoked by IT-system breakdown are modulated dynamically on a behavioral and neuronal level, we performed an fMRI experiment during which participants solved arithmetic problems of different difficulty levels while system runtime errors were inserted in an unpredictable manner during the problem presentation or the feedback phase following the participant’s response. Our results showed that participants’ performance was positively influenced for the hard tasks when a runtime error occurred at the problem presentation phase, while their performance was adversely affected on the hard tasks when an error occurred during the feedback phase. Moreover, blood-oxygen-level-dependent (BOLD) signals showed that the default mode network played a significant role in participants’ modulation of performance. These results shed light upon the decision-making and neuronal modulation processes underlying people’s responses to IT system breakdowns.

## Methods

### Participants

A total of 15 healthy young adults (10 female, 5 male; mean age 24.4 yrs; age range from 19 to 31 yrs) participated in the study. The number of participants is comparable to that used in previous studies investigating technostress using neuroimaging methods^17,21,44^. All participants were pre-screened for psychiatric and neurological disorders and were not taking psychoactive medication at the time of study. All participants were evaluated to meet the criteria for MRI safety and they had normal or corrected-to-normal vision. All but one were right-handed. Participants received monetary compensation. Prior to the experiment, participants were told that their compensation was contingent on the number of correct answers recorded during the session. However, all participants received the full (maximum possible) compensation of 14.40 Euro after the experiment. Participants gave written informed consent before participating in the study. The study was approved by the ethics committee at the University of Regensburg in accordance with the Declaration of Helsinki.

### Experimental procedure

**Figure 1:**
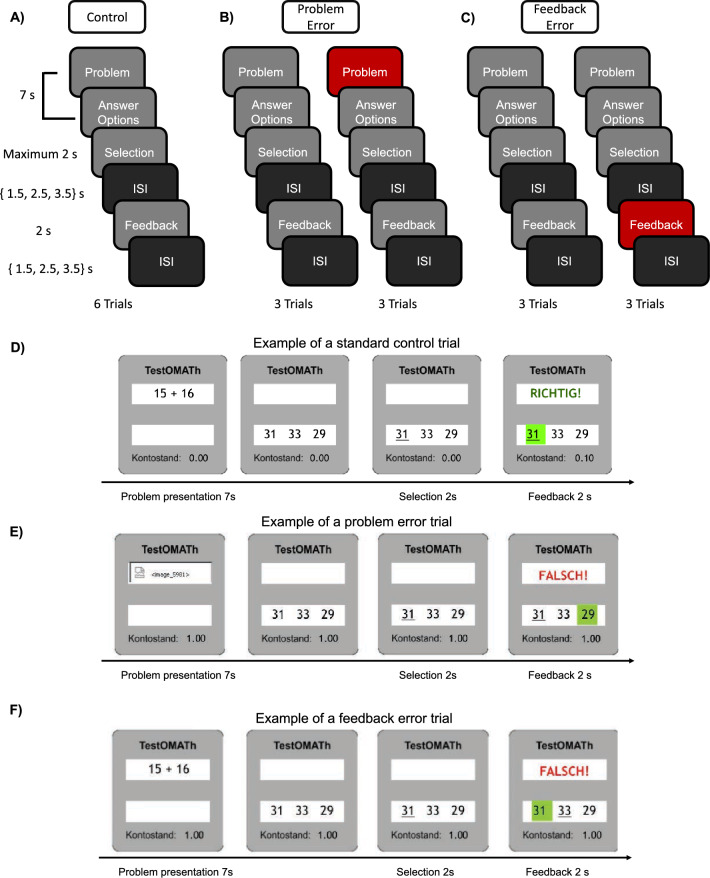
Experimental procedure. Arithmetic problems of three difficulty levels were presented in 3 sub-blocks corresponding to 3 conditions: (**A**) The control condition. Each block consisted of 6 trials. Each trial consisted of a 7-s problem presentation phase, 2-s selection phase, a 2-s feedback phase, an ISI of 1.5, 2.5 or 3.5 secs before and after the feedback phase. (**B**) The problem error condition. Same as (**A**), but 3 trials in the condition contained a problem error. (**C**) The feedback error condition. Same as (**A**), but 3 trials in the condition contained a feedback error. (**D**) Example of a standard control trial without errors. Participants’ response choices were underlined in the response selection stage. The correct responses were highlighted in green along with the feedback (the German words ‘Richtig’ corresponds to correct in the upper panel and ‘FALSCH’ corresponds to false in the lower panel). Participants’ accumulated reward for each correct trial was presented throughout the trials (shown in ‘Kontostand’). (**E**) Example of a trial with a problem error. A system error image was presented during the problem presentation stage. Otherwise, the same as (**D**). (**F**) Example of a trial with a feedback error. The system falsely repeated the participants’ choices and provided the wrong feedback. Otherwise, the same as (**D**).

Participants were requested to solve additive arithmetic problems of three difficulty levels throughout the experiment. The easy problems required adding two two-digit numbers, the moderately challenging (medium) problems involved adding one two-digit and one three-digit number, and the hard problems required adding two three-digit numbers. A standard trial (control condition) consisted of three phases: the problem presentation phase, the response selection phase, and the feedback phase. Each trial began with a 7-s problem presentation phase (5 secs problem presentation and 2 secs response option presentation) followed by a 2-s response selection phase, in which participants were instructed to choose one option from three alternatives by pressing the corresponding button with their index, middle or ring finger (Fig. 1A). The feedback phase followed the response selection phase after a jittered inter-stimulus-interval (ISI) of 1.5, 2.5 or 3.5 secs and lasted 2 secs. During the feedback phase, the participants' choice was shown together with the correct answer. Each trial commenced with a blank inter-trial-interval (ITI) that was also jittered in duration, i.e., of either 1.5, 2.5 or 3.5 secs. The use of this temporal jitter increased the statistical separation of the effects of the regressors entered into the general linear model (GLM). The length of the intervals was pseudorandomly selected and counterbalanced across the trials and across participants. Participants were offered a monetary bonus (10 cent) as a reward for each correctly solved problem and the accumulated reward amount (i.e., bonus) was constantly displayed throughout the trials (Fig. 1D).

We included the unreliability of the IT system as a potential source of technostress. Unreliability was operationalized by two different system malfunctions. Some trials had either a problem error or a feedback error. During the problem error trials, participants were presented with an image indicative of a system error in the computer program (Fig. 1B,E), which made the numbers illegible. After the presentation of the error image, the response options were presented normally as if no error had occurred. Participants were instructed to attempt to solve each problem and they received feedback as in the control condition. During feedback-error trials (Fig. 1C,F), the program showed the correct answer to the problem, but falsely repeated the choice of the participant in the feedback phase suggesting that the program misrecorded the participant's choice. The trial was counted incorrect even when participants had responded correctly (Fig. 1F).

The experiment consisted of 8 blocks separated into two runs, where each block consisted of 3 sub-blocks corresponding to 3 conditions: the control condition, the problem error condition and the feedback error condition (see, Fig. 1A–C). A 18 s baseline period was presented between the blocks. Participants solved a total of 6 arithmetic problems of all the three difficulty levels for each sub-block. Half of the trials (3 trials) in the problem error sub-block contained an error during the problem presentation phase as shown in Fig. 1B. Half of the trials (3 trials) in the feedback error condition consisted of a feedback error (Fig. 1C). In total 18 trials (3 sub-blocks × 6 trials) made up a block and 72 trials (4 blocks × 18 trials) made up a run and the first and second run were separated by a brief pause in MRI data acquisition. The order of the difficulty levels and the order of the conditions were counterbalanced and pseudorandomized for each block and across the participants.

Participants practiced the task for two repetitions of the control condition (with no errors) before the experiment started and they were not informed that an error might occur during the experiment. Finally, a high-resolution structural scan lasting about 10 min was conducted after completion of the two fMRI runs.

### Apparatus

The experiment was controlled and presented via the Presentation^®^ software (Neurobehavioral Systems, Inc., Berkeley, CA, www.neurobs.com). The visual display was projected onto a translucent screen located in the scanner via a PROPixx projector (VPixx Technologies, Saint-Bruno-de-Montarville, Canada). The projected image had a resolution of 1024 × 768 pixels with a refresh rate of 60 Hz. Participants viewed the visual display via a headcoil-mounted mirror. Participants made their responses with an MRI-compatible button box (Psychology Software Tools, Pittsburgh, PA, United States).

### MRI parameters

All participants were scanned in a 3 T Siemens Prisma scanner (Siemens Healthineers, Erlangen, Germany) at the University of Regensburg with a 64-channel head coil. A T1-weighted MPRAGE sequence (176 sagittal slices, field of view: 256 × 240 mm^2^, voxel size: 1 × 1 × 1 mm^3^, repetition time: 2300 ms, echo time: 2.98 ms, flip angle: 9°) was acquired for each fMRI session. The parameters were adapted from the Alzheimer's disease Neuroimaging project (Laboratory for Neuro Imaging, UCLA, Los Angeles, CA). Functional MRI runs were acquired with a multi-band T2*-weighted gradient-echo sequence with echoplanar read-out (repetition time: 2000 ms, echo time: 30 ms, flip angle: 90°, voxel size: 2 × 2 × 2 mm^3^, field of view: 212 × 204 mm^2^, multi-band factor: 2) along 64 axial slices covering the whole brain.

### Data processing

#### Behavioral data processing

Participants’ response accuracy data were recorded and analyzed in the fMRI experiment. First, we averaged participants’ response accuracy for each of the three difficulty levels and for each condition separately for each experimental run. The trials in which a problem error was presented were not included in the calculation of each participant’s accuracy. The trials before the occurrence of the first error trials in the problem error and the feedback error condition were excluded from the accuracy calculation. ~ 3.66 trials were excluded for each participant. Moreover, in order to balance the number of trials for different conditions, we randomly selected trials in the control and the feedback error conditions for 10,000 times and calculate the accuracy scores by averaging the random selected trials. This calculation yielded 9 accuracy scores (3 conditions × 3 difficulty levels) for each block. Secondly, we averaged the individual accuracy score according to two different time intervals: the first experimental run (the 1st–4th block) and the second experimental run (the 5th–8th block). The time intervals were chosen such that we were capable of detecting participants’ performance and strategy change during the evolution of the session. We hypothesized that participants would be the most surprised during the first interval and slowly adjusted to the occurrence of errors during the first half of the session (first run). During the second half of the session (second run), participants’ performance should become stabilized as they get 'accustomed' to the runtime errors.

### fMRI data processing

#### Preprocessing

The structural and functional MRI runs were preprocessed using the Freesurfer Software package (Version 4.5) and the FSFast toolbox^45,46^ together with Matlab (version R2020b, The MathWorks Inc., Natick, MA, United States). The high-resolution structural run was reconstructed and inflated for each participant as described in previous studies^47^. The images from the fMRI runs were motion-corrected and realigned to the first volume of the first run, based on which a template was created. The template was co-registered to the reconstructed high-resolution structural scan of each individual participant. The images from the fMRI runs were then slice-time corrected, intensity normalized, and spatially smoothed with a three-dimensional Gaussian kernel (full-width-half-maximum: 5 mm).

#### Analysis of fMRI runs

To examine the change in BOLD responses that were specifically related to participants’ decision-making process, which corresponds to the problem presentation phase, we analyzed the preprocessed signals in the fMRI runs in an event-related manner for the first level analysis. Two general linear models (GLM) assuming a gamma-shaped hemodynamic response function (HRF) with a delay of 2.25 s and a dispersion of 1.25 s were fitted separately for each preprocessed fMRI run so that the BOLD signal analyses shared similar time intervals as in the behavioral data processing. The GLM included 6 regressors-of-interest as well as nuisance regressors-of-no-interest corresponding to motion correction parameters and linear scanner drift. The 6 regressors-of-interest corresponded to the problem presentation phase of the three types of trials (standard control trial, with problem error, and with feedback error). The additional 3 regressors-of-interest corresponded to the feedback phase of the three types of trials. The signals during the selection stage and the standard trials before the first occurrence of error trials in the problem error and feedback error condition were fitted as two additional regressors-of-no-interest to obtain the BOLD responses targeting the processes underlying participants’ problem-solving phase. Contrasts (problem error versus feedback error; problem error versus control) were obtained for each subject. For the second level analysis, the contrasts were concatenated to calculate the group-level significance through one-sample t-tests on the voxels of the whole brain. Multiple comparisons were corrected for false discovery rate at *p* = 0.01. The BOLD activations were extracted from each of the ROIs (see *Definition of ROIs*), averaged across the two hemispheres and normalized to the activation baseline to acquire the BOLD percent signal change.

#### Definition of ROIs

**Figure 2:**
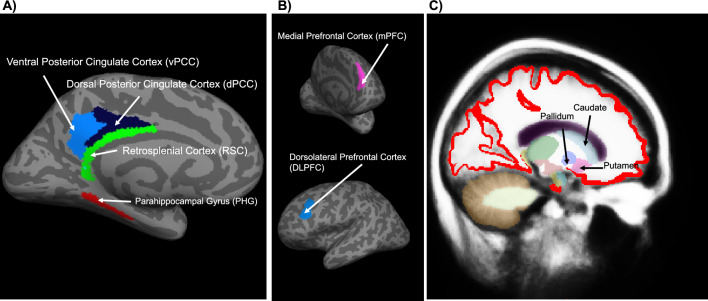
Location of ROIs on the left hemisphere of a representative participant. (**A**) ROIs of the default mode network on the inflated left hemisphere of the representative participant (dark gray: sulci; light grey: gyri) (**B**) mPFC (the anterior view) and DLPFC (the medial view) on the inflated left hemisphere. (**C**) subcortical ROIs of the executive network (sagittal view, red = gray matter, other colors = other subcortical regions that are not analyzed in our study).

We selected ROIs that are mainly involved in the default mode network with the posterior cingulate cortex as a hub^48,49^: the dorsal posterior cingulate cortex (dPCC;^43,50^), the ventral posterior cingulate cortex (vPCC;^32^), the retrosplenial cortex (RSC), the parahippocampal gyrus (PHG) and the medial prefrontal cortex (mPFC). We also selected ROIs that are mainly involved in the executive network^48^: the dorsolateral prefrontal cortex (DLPFC), the dorsal striatum (caudate and putamen), and the ventral striatum (pallidum). It has been suggested that the inferior and superior parietal cortices^51,52^ as well as the angular and supermarginal gyri^53^ are involved in the processes of numerical computation itself. To avoid confounding the numerical computation and the processes underlying the modulation of unpredicted technical errors, we did not select the above-mentioned cortices in the ROI analyses. We defined each participant’s cortical ROIs by using the parcellation of the human cerebral cortex proposed by Glasser and colleagues^54^. The subcortical nuclei were defined using the ‘aseg’ segmentation from Freesurfer^55,56^. Specifically, we have delineated the dorsal PCC using the areas 23d, d23ab, 31a of the Glasser atlas. The ventral PCC consisted of area v23ab, area 31 pd and area 31pv. These ROIs of the PCC correspond to those in previous publications^32,37,43,50,60^. The ROIs mPFC and DLPFC were delineated with the area 9a and 46 respectively. The MNI coordinates of the center of mass of the ROIs are described in Table 1. The locations of the ROIs of a representative participant are shown in Fig. 2.

**Table 1:** Description of the regions-of-interest analyzed in this study.

		X	Y	Z	Size
The default mode network	Left dPCC	− 3 ± 2.7	− 20 ± 12.5	33 ± 10.8	995 ± 139
	Right dPCC	7 ± 3.0	− 20 ± 12.7	34 ± 10.7	1009 ± 116
	Left vPCC	− 7 ± 3.1	− 35 ± 12.1	24 ± 11.9	1203 ± 139
	Right vPCC	2.3 ± 3.2	− 35 ± 12.0	25 ± 11.6	1276 ± 120
	Left RSC	− 5 ± 2.7	− 20 ± 11.6	20 ± 11.0	602 ± 69
	Right RSC	7 ± 2.5	− 18 ± 11.4	21 ± 10.9	667 ± 60
	Left PHG	− 22 ± 3.4	− 16 ± 11.4	− 9 ± 11.1	466 ± 48
	Right PHG	20 ± 3.2	− 14 ± 10.4	− 11 ± 10.7	375 ± 45
	Left mPFC	− 8 ± 3.0	61 ± 12.7	36 ± 9.4	1169 ± 147
	Right mPFC	8 ± 2.7	62 ± 12.0	33 ± 9.5	1477 ± 128
The executive network	Left DLPFC	− 32 ± 3.0	44 ± 12.8	40 ± 10.1	1311 ± 177
	Right DLPFC	32 ± 3.4	46 ± 12.0	37 ± 10.1	1402 ± 182
	Left Caudate	− 11 ± 2.2	23 ± 11.7	17 ± 9.9	3788 ± 402
	Right Caudate	12 ± 1.8	25 ± 11.6	17 ± 9.7	3959 ± 398
	Left Pallidum	− 19 ± 1.8	12 ± 11.0	6 ± 10.2	2098 ± 179
	Right Pallidum	19 ± 1.7	13 ± 11.0	5 ± 10.0	1942 ± 154
	Left Putamen	− 24 ± 2.0	17 ± 11.5	8 ± 10.1	5345 ± 677
	Right Putamen	24 ± 1.8	19 ± 11.0	7 ± 9.8	5474 ± 506

MNI coordinates (X, Y, Z) show the mean center of mass position in native space across all participants. In addition, standard deviations are shown. The size is listed in voxels (8 mm^3^) in the native space.

#### Granger causality (GC) analysis

In order to further investigate how the five ROIs (dPCC, vPCC, RSC, mPFC and PHG) involved in the default network influenced each other with the dPCC as a hub, effective connectivity was examined using the multivariate Granger causality toolbox^57^. This analysis allowed us to describe how the BOLD time series in one ROI influenced the BOLD time series in another ROI in the selected network of ROIs^58–60^.It is assumed that the time series that causes changes in other time series must precede and assist the prediction of the other time series. For instance, if the past in the time series in PCC contains information that enhances the prediction of the future of the time series in RSC, in addition to the information that has already existed in the past of the time series in RSC, PCC can be described as ‘Granger-causing’ RSC^61^. Thus, to measure such relationships in the default mode network, we performed paired-wise GC analysis among the three ROIs. First, we preprocessed the first fMRI run with motion-correction, slice-time correction, intensity normalization and spatial smoothing with a three-dimensional Gaussian kernel (full-width-half-maximum: 5 mm). Second, we computed variance-of-no-interest which consists of the white matter, ventricles and the cerebrospinal fluid. Third, a GLM was fitted to the fMRI time series with this variance-of-no-interest as well as motion correction parameters and linear scanner drift as nuisance regressors. Fourth, we extracted the residual of the GLM from each of the five ROIs. The residual corresponds to the fMRI time series with these sources of variance-of-no-interest accounted for and removed from further analyses. Fifth, we averaged the residual across the voxels in the five ROIs. These time-series data included the signals from all of the experimental stages, as the GC analysis required continuous time-series data. Finally, we fitted multivariate autoregressive models (MVAR) to the averaged time-series signals, which were used as inputs to compute the GC coefficients. The GC coefficients were Fisher-transformed, and the significance of the GC coefficients was multiple-corrected by controlling the false discovery rate (FDR) at *p* = 0.05.

### Statistical analyses

Statistical analyses were conducted using Matlab (R2020b) and the SPSS software (IBM; version 24). The normality of the data was tested with the Shapiro–Wilk test. All the data were normally distributed, and parametric statistical tests were thus conducted. ANOVAs were conducted for within-group comparisons between different conditions, and post-hoc tests were performed with two-tailed paired sample t-tests. Effect sizes were reported as partial *η*^*2*^ and Cohen’s d, respectively, for ANOVAs and t-tests. Multiple comparisons were corrected by controlling the false discovery rate for the behavioral, neural and Granger causality analyses. The significance level was set at *p* = 0.05 for the behavioral, ROI and Granger causality analyses. The significance level for the group-level whole-brain analyses was set at *p* = 0.01. Corrected p-values are reported below.

## Results

### Behavioral data

**Figure 3:**
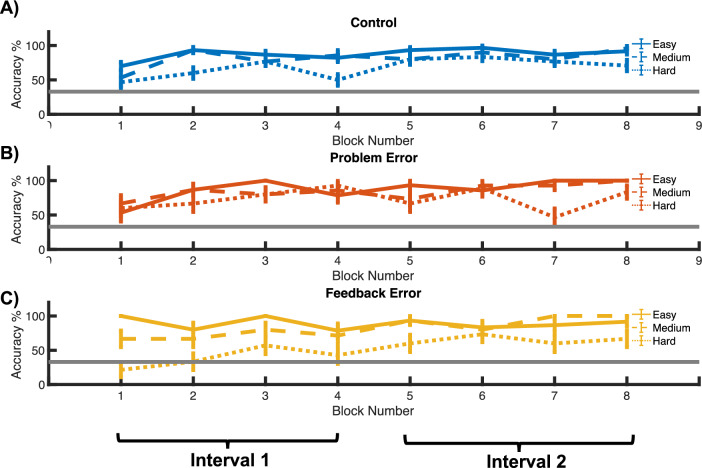
Behavioral performance for each block for (**A**) the control condition (blue), (**B**) the problem error condition (orange), (**C**) the feedback error condition (yellow). The easy problems are represented with solid lines, the moderately challenging (medium) problems with dashed lines, and the hard problems with dotted lines. The 8 blocks of 18 trials each were separated into two time intervals: the 1st–4th block, and 5th–8th block as the first and second intervals. The solid gray line represents chance level response at 33.3%.

Participants’ accuracy in performing the arithmetic operation was obtained separately for each of the difficulty levels (easy, medium and difficult), for each condition (control, problem error and feedback error), and for each run separately (Fig. 3) after removing the trials with a problem error, trials preceding an error in the problem and feedback error conditions and the number of trials balanced across conditions (see “Methods” section for details).

**Figure 4:**
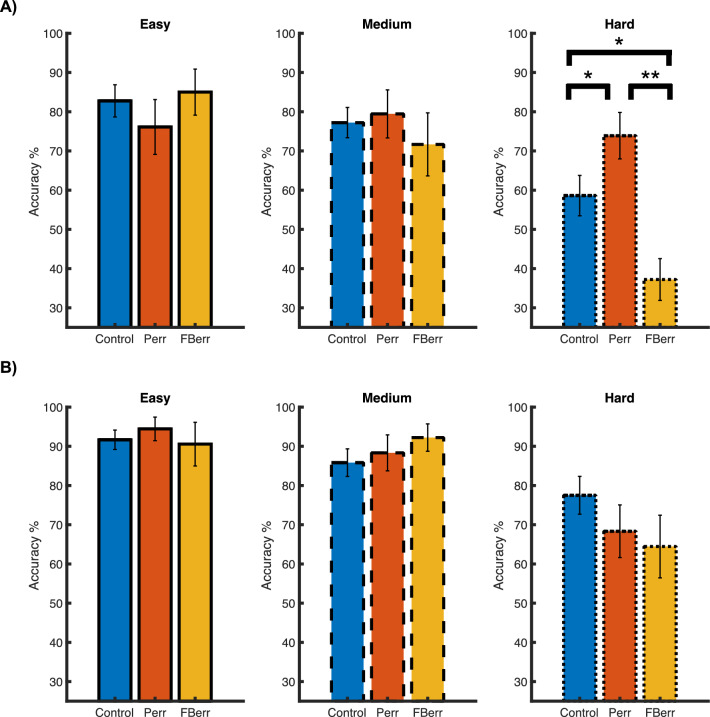
Behavioral performance for two different time intervals. In panels (**A**) and (**B**) the accuracy scores for the first and second time-interval are presented, respectively. Different colors represent the three conditions, as in Fig. 3. The three difficulty levels are presented in separate panels, and the results are highlighted by solid, dashed and dotted outlines as in Fig. 2. *p < 0.05. *Perr* problem error condition, *FBerr* feedback error condition.

In order to better evaluate how participants’ performance evolved over time, we separated participants’ accuracy scores into two time intervals (see “Methods” for details): the 1st–4th block (the first fMRI run) and the 5th–8th block (the second fMRI run) (Fig. 4A,B). For each time interval, we performed a repeated-measure ANOVA for each difficulty level with ‘Condition’ as the within-subject factor. During the first interval, the ANOVA yielded a significant difference only at the hard level [*F*(2, 28) = 5.286, *p* = 0.033, partial *η*^*2*^ = 0.274] and no significant difference at the other two difficulty levels [*F*(2, 28) = 2.667, *p* = 0.130] and [*F*(2, 28) = 0.056, *p* = 0.945] as shown in Fig. 4A. Post-hoc t-tests showed that the accuracy score for the problem error condition was significantly higher than the feedback error condition[*t*(14) = 3.492, *p* = 0.011, Cohen’s d = 0.902]. The accuracy score for the problem error condition did not differ significantly from the control condition [*t*(14) = 1.871, *p* = 0.203]. Moreover, the accuracy score for the feedback error condition was not significantly different from that in the control condition [*t*(14) = 1.333, *p* = 0.203]. The ANOVAs during the second time interval yielded no significant differences among the different conditions over all three difficulty levels, as shown in Fig. 4B [all *p*s > 0.05].

Furthermore, we evaluated whether participants’ performance changed from the first to the second interval for each condition. We found a significant performance improvement on hard trials for the control [*t*(14) = 2.348, *p* = 0.049, *Cohen ’d* = 0.610] and the feedback error [*t*(14) = 3.341, *p* = 0.014, *Cohen ’d* = 0.863] conditions. Moreover, participants’ performance remained unchanged over time for the problem error condition [*t*(14) = 0.756, *p* = 0.462]. Additionally, participants improved on the medium-difficult trials for the control condition as well [*t*(14) = 2.870, *p* = 0.011, *Cohen ’d* = 0.903].

### fMRI data

#### Whole-brain analyses

**Figure 5:**
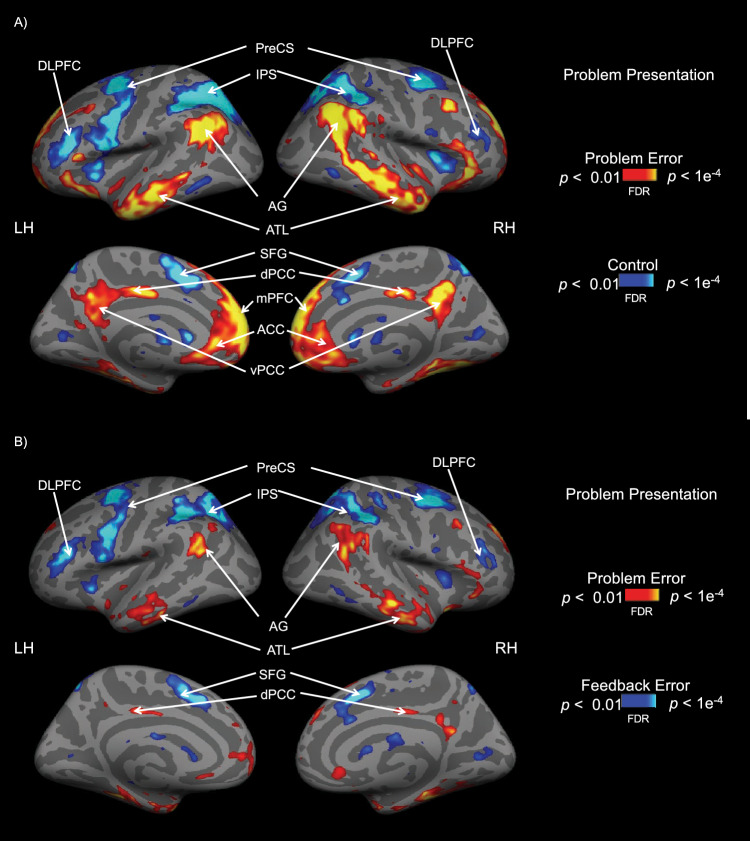
Whole-brain group analyses during the first time interval for the contrast problem error (**A**) versus control and problem error versus feedback error (**B**). FDR corrected at* p* = 0.01. *DLPFC* dorsolateral prefrontal cortex, *PreCS* precentral sulcus, *IPS* intraparietal sulcus, *AG* angular gyrus, *ATL* anterior temporal lobe, *SFG* superior frontal gyrus,*dPCC* dorsal posterior cingulate cortex, *vPCC* ventral posterior cingulate cortex, *mPFC* medial prefrontal cortex, *ACC* anterior cingulate cortex.

To describe the brain activations between the different conditions on a group level, we performed a whole-brain analysis for the first and second time interval with the following contrasts: problem error versus control, problem error versus feedback error and feedback error versus control. The whole-brain analysis showed significant contrasts only during the first interval. The brain activations for the contrasts, problem error versus control and problem error versus feedback error were shown in Fig. 5A and B. There was no significant brain activation difference for the contrast feedback error versus control. For the problem error condition, there were significant neural activities in the dorsal and ventral posterior cingulate cortex, the medial prefrontal cortex, the angular gyrus, and the anterior and middle temporal gyrus. The neural activations are more pronounced in the dorsolateral prefrontal cortex, intraparietal sulcus and the precentral sulcus for the feedback error and the control conditions.

#### BOLD activations in the default mode network

**Figure 6:**
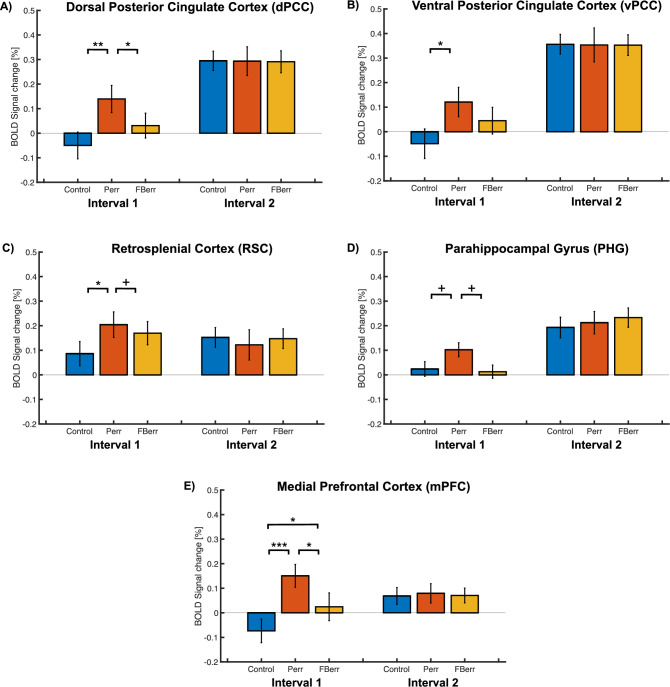
BOLD percent signal change for two different time intervals in the five ROIs in the default mode network. In panels (**A**–**E**), the BOLD percent signal change for the dPCC, the vPCC, the RSC the PHG and the mPFC are presented. The color codes and x-axis labels are the same as in Fig. 4. Difficulty levels are collapsed within each condition*.* ^+^*p* < 0.01, *p < 0.05, **p < 0.01, ***p < 0.001.

To measure the differences in BOLD activations between the different conditions, we performed a one-way repeated-measures ANOVA with ‘Condition’ separately for each of the two time intervals in the dPCC, vPCC, the retrosplenial cortex (RSC), and the parahippocampal gyrus (PHG) and the medial prefrontal cortex (mPFC). During the first time interval (the 1st to 4th block), there was a significant difference in both the dorsal [*F*(2, 28) = 10.709, *p* = 0.002, partial *η*^*2*^ = 0.433] and ventral PCC [*F*(2, 28) = 9.005, *p* = 0.003, partial *η*^*2*^ = 0.391] among the three levels of difficulty. Post-hoc t-tests in the dPCC revealed that the BOLD percent signal changes in the problem error condition were significantly higher than that in the control [*t*(14) = 4.800, *p* < 0.001, Cohen’s d = 1.240] and the feedback error condition [*t*(14) = 2.556, *p* = 0.035, Cohen’s d = 0.660] condition (Fig. 6A). There was significant BOLD percent signal change difference between the problem error and the control condition [*t*(14) = 24.493, *p* = 0.001, Cohen’s d = 1.160] in the vPCC (Fig. 6B). Significant within-subject effects were found during the first time-interval in both the ROIs: the RSC, [*F*(2, 28) = 6.224, *p* = 0.006 partial *η*^*2*^ = 0.308] and the mPFC [*F*(2, 28) = 18.397, *p* < 0.001, partial *η*^*2*^ = 0.568]. A marginal significant effect of ‘Condition’ was found in the PHG [*F*(2, 28) = 3.867, *p* = 0.059, partial *η*^*2*^ = 0.216]. During this time interval, the RSC showed significantly higher BOLD percent signal change in the problem error condition [*t*(14) = 3.570, *p* = 0.009, Cohen’s d = 0.921] in comparison to the control condition (Fig. 6C). Post-hoc t-tests in the mPFC revealed that the BOLD percent signal changes in the problem error condition were significantly higher than that found in the control [*t*(14) = 6.824, *p* < 0.001, Cohen’s d = 1.762] and in the feedback error conditions [*t*(14) = 2.832, *p* = 0.013, Cohen’s d = 0.731], and the feedback error condition has a higher BOLD percent signal change compared to the control condition [*t*(14) = 2.974, *p* = 0.013, Cohen’s d = 0.768] (Fig. 6E). Additionally, the BOLD percent signal change of the problem error condition is marginally higher than that of the control condition in the PHG *t*(14) = 2.239, *p* = 0.063, Cohen’s d = 0.578] and marginally higher than that of the feedback error condition in the RSC *t*(14) = 2.420, *p* = 0.063, Cohen’s d = 0.591] and the PHG *t*(14) = 2.448, *p* = 0.063, Cohen’s d = 0.632]. Similar to the behavior analysis, the ANOVAs performed during the second time interval in the dPCC, the other ROIs in the default mode network did not reveal any significant within-subject effects (Fig. 6, all *p*s > 0.05). We found no correlations between the differences in BOLD activations in the problem-error and feedback-error conditions and the differences in behavior performances for the respective conditions.

#### BOLD activations in the executive network

**Figure 7:**
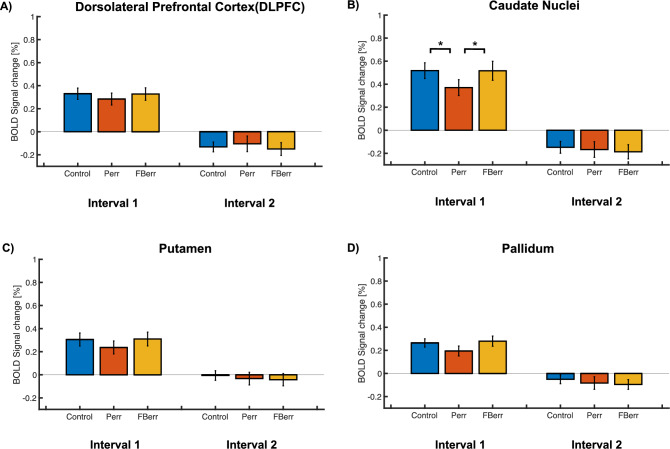
BOLD percent signal change for two different time intervals in the ROIs in the executive network. Each figure (**A**–**D**) represents the BOLD percent signal change for the DLPFC, the caudate nuclei, the putamen, and the pallidum. Otherwise, the same as Fig. 6.

We performed a one-way repeated measures ANOVA for the ROIs of the executive network as for the posterior cingulate network. The within-subject effect was only significant during the first time interval for the caudate nuclei [*F*(2, 28) = 4.277, *p* = 0.002, partial *η*^*2*^ = 0.450] (Fig. 7A). In contrast to the BOLD activations in the PCC, the BOLD percent signal change in the problem error condition was significantly lower than that in the control condition [*t*(14) = 4.277, *p* = 0.002, Cohen’s d = 1.104] and the feedback error condition [*t*(14) = 3.890, *p* = 0.003, Cohen’s d = 1.00]. Although the ANOVA was significant for the DLPFC [*F*(2, 28) = 8.290, *p* = 0.003, partial *η*^*2*^ = 0.371], post-hoc tests did not reveal significant differences across conditions. The other ANOVAs performed for the DLPFC, the putamen and the pallidum, as well as the ANOVAs during the second time interval in the caudate, did not show any significant effects (Fig. 7B–D). We found no correlations between the differences in BOLD activations in the problem-error and feedback-error conditions and the differences in behavior performances for the respective conditions.

#### BOLD activation differences between time intervals

To assess whether the BOLD percent signal changed from the first to the second time interval similarly as that found for the behavioral performance, we conducted pair-wise t-tests for each condition in each of the selected ROIs. The BOLD percent signal change was enhanced in the ROIs: dPCC, vPCC for all of the three conditions. The BOLD activations were enhanced in the PHG for the control and feedback error conditions. Moreover, the BOLD percent signal change decreased for all of the ROIs in the executive network (DLPFC, caudate nuclei, putamen and pallidum). The statistics and p-values for the above mentioned comparisons were summarized in Table 2.

**Table 2:** Statistics and p-values for the significant difference of BOLD percent signal change across time intervals.

		t(14)	*p*	Cohen’d
The default mode network	dPCC—Perr	2.381	0.041	0.615
	dPCC—FBerr	3.808	0.004	0.983
	dPCC—Control	4.180	0.002	1.079
	vPCC—Perr	2.667	0.026	0.689
	vPCC—FBerr	4.469	0.002	1.154
The executive network	vPCC—Control	3.683	0.002	0.951
	PHG—FBerr	4.259	0.002	1.099
	PHG—Control	2.776	0.022	0.716
	DLPFC—Perr	3.865	0.004	0.998
	DLPFC—FBerr	5.530	< 0.001	1.430
	DLPFC—Control	4.810	< 0.001	1.241
	Caudate—Perr	4.820	0.005	1.244
	Caudate—FBerr	5.824	< 0.001	1.504
	Caudate—Control	6.299	< 0.001	1.628
	Putamen—Perr	3.377	0.006	0.872
	Putamen—FBerr	4.076	0.002	1.053
	Putamen—Control	4.213	0.002	1.088
	Pallidum—Perr	4.165	0.002	1.075
	Pallidum—FBerr	5.686	< 0.001	1.468
	Pallidum—Control	5.795	< 0.001	1.496

*Perr* Problem error, *FBerr* feedback error.

p-values are FDR-corrected.

#### Granger causality (GC) in the posterior cingulate network

**Figure 8:**
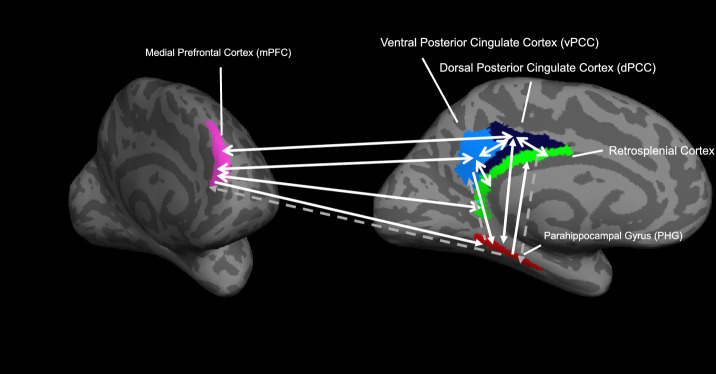
Granger causality (GC) in the posterior cingulate network among the dorsal and ventral posterior cingulate cortex (dPCC and vPCC), the retrosplenial cortex (RSC), the medial prefrontal cortex and the parahippocampal cortex (PHG). Solid white arrows represent significant GC, and dashed gray arrows represent non-significant GC. The direction of the arrow represents the direction of the ‘granger-causal’ influence. The colors represent different ROIs, as in Fig. 2A and B. The ROIs are displayed on frontal and medial views of the left hemisphere of the inflated brain of a representative participant.

The BOLD analyses indicated that the default mode network with the posterior cingulate as a hub might play a more pronounced role in modulating participants’ strategies throughout the task. In order to describe the interactive relationships within this network, we performed GC analysis. The Fisher transformed GC coefficient of the region ‘granger-causing’ the activities in the other ROIs are reported in parentheses below. The analysis (Fig. 8) revealed significant pairwise GC among the BOLD time courses in dPCC (vPCC: Fisher-transformed GC coefficient: 0.019, *p* < 0.0001; mPFC: Fisher-transformed GC coefficient: 0.014, *p* < 0.0001), vPCC (dPCC: Fisher-transformed GC coefficient: 0.001, *p* = 0.019; mPFC: Fisher-transformed GC coefficient: 0.0105, *p* < 0.0001) and mPFC (dPCC: Fisher-transformed GC coefficient: 0.015, *p* < 0.0001; vPCC: Fisher-transformed GC coefficient: 0.011, *p* < 0.0001). These three regions also granger-causally influence the time courses both the PHG (dPCC: Fisher-transformed GC coefficient: 0.001, *p* = 0.001; vPCC: Fisher-transformed GC coefficient: 0.003, *p* < 0.0001; mPFC: Fisher-transformed GC coefficient: 0.002, *p* < 0.0001) and the RSC (dPCC: Fisher-transformed GC coefficient: 0.001, *p* = 0.008; vPCC: Fisher-transformed GC coefficient: 0.016, *p* < 0.0001; mPFC: Fisher-transformed GC coefficient: 0.007, *p* < 0.0001). Moreover, the BOLD time courses in the PHG additionally ‘Granger-causes’ those in the RSC (Fisher-transformed GC coefficient: 0.004, *p* < 0.0001) and dPCC (Fisher-transformed GC coefficient: 0.002, *p* < 0.0001). The BOLD time courses in the RSC causally influence the activities in dPCC (Fisher-transformed GC coefficient: 0.001, *p* = 0.01), the vPCC (Fisher-transformed GC coefficient: 0.001, *p* < 0.0001) and the mPFC (Fisher-transformed GC coefficient: 0.004, *p* < 0.0001). The GC analyses in the other directions between these regions did not show significant relationships, all *p*s > 0.05 after FDR correction.

## Discussion

In this study, we examined how unpredicted IT system errors dynamically influence people’s performance in solving problems of varying difficulty levels, as well as the underlying neural mechanisms that modulate such processes. Surprisingly, we found that an unpredicted error at the problem presentation phase could positively influence participants’ performance at the most difficult task level from the initial stage of the experiment. On the other hand, the influence of an error during the feedback phase on participants’ performance was negative for the hard arithmetic tasks during the first stage of the experiment. Furthermore, while a problem error could immediately affect participants’ performance and be coped with in an early stage of the experiment, the effect of a feedback error evolved over time. Participants’ performance recovered from the influence of feedback errors at a later stage of the experiment. One possible explanation for this difference is that problem errors are more salient and easier to detect than feedback errors. Moreover, participants could rely more extensively on feedback when they were solving hard arithmetic problems compared to when they were solving easy or moderately challenging arithmetic problems, in which self-generated feedback provided sufficient confidence in their decision-making processes. In association with the observed dynamic behavior change, we found that the default mode network, including the dorsal and ventral PCC, the mPFC, the PHG and the RSC rather than the executive network, is largely involved in the modulation of participants’ performance.

Coinciding with the time intervals in which performance was enhanced for the problem and feedback error condition, the BOLD percent signal changes in the default mode network with the PCC as a hub increased during the 1st–4th blocks only in the problem error condition, when the error was initially detected and coped with by the participants. At the second time interval (5th–8th block), when the performance for feedback error and the control condition was enhanced, the BOLD signal percent changes in the default mode network increased accordingly in both the control and the feedback error condition. Unlike in the default mode network, the neural activities in the executive network play a less important role for the dynamic neural modulation processes. During the first time interval, there was a decrease of BOLD percent signal change for the problem error condition only in the caudate nuclei. During the second time interval, the BOLD percent signal changes for all of the ROIs in the executive network were close to baseline level.

The default mode network appears to modulate participants’ dynamic responses to unpredicted technical errors through the activation and coactivation of different highly connected brain regions. The dPCC, vPCC and the mPFC appear to be the hub of the network, with extensive connections with the RSC and PHG. While the RSC is closely connected with the vPCC, the PHG is mainly associated with the dPCC. The ‘causal’ relationship of BOLD activations among these regions, as described by the GC causality analysis (see Fig. 8), suggests a similar dependency among the three regions, with the PCC and mPFC serving as the network controller.

Our finding is in agreement with recent theories about the cognitive functions of the default mode network and the posterior cingulate cortex that have been established using both human and animal models^32,38,39,41–43,62–64^. The neuronal activities in the posterior cingulate cortex can reflect the detection of rapid changes in the environment (in our case, unpredicted runtime errors) as well as change in the context of the demanding task (e.g. the block in which the error occurred) to promote the search for new strategies and policies to maximize participants’ behavioral outcomes. It is also worth noting that both the behavioral accuracy and neuronal activities in the PCC and PHG were also enhanced for the control condition as the experiment proceeded, suggesting that the PCC and PHG might not only be involved in the processing of unexpected errors but also in evaluating and generalizing their strategies across conditions (context) in the experimental environment. The roles of the PCC and PHG might be involved in evaluating the task environment, adjusting strategies and allocating resources to perform the task across trials and conditions^37,38,43^. As participants progressed through the experiment, they could familiarize themselves with the timing of when an error might occur. Consequently, they have reallocated their resources to prioritize their performance of the task when no errors would occur, in particular for the control condition. Accordingly, we observed performance enhancement for the control condition on both the medium and hard trials.

The lack of modulation from the executive control network might be related to how the error conditions have been constructed in our study. The occurrence of errors in our study were predictive of the blocks or context the participants were experiencing. The occurrence of errors did not provide information about the upcoming trials, which would have allowed the participants to conduct a trial-by-trial update of the information they have accumulated over the course of performing the arithmetric tasks as done in many previous studies^27,29,42^. It is possible that the change in environmental context are more closely related to the PCC and the default mode network rather than the DLPFC and the basal ganglia nuclei in the executive control network.

While the design of our study has allowed us to measure the neuronal and behavioral responses to unpredicted runtime errors, we could not determine how these neuronal changes are related to participants’ stress states. Thus, our study could benefit from additional physiological measurements, including heart rate and cortisol levels, to quantitively measure the corresponding stress responses of the participants. Our study would also benefit from psychophysiological correlations between the trial-by-trial performance of the participants and the pattern of BOLD responses they exhibit in the above-described cortical networks.

One potential limitation of our study is the small number of participants. Although similar to previous publications, the statistical power of the results and the generalizability of our interpretation of the study could be significantly improved with a larger sample size.

To conclude, our study suggests that the posterior cingulate cortex system is largely involved in modulating the dynamic behavioral outcomes in response to unpredicted technical errors in human–computer interactions (i.e., technostress arising from techno-unreliability). Our study helps promote the understanding of how technical breakdowns of pervasive information technologies might be associated with stress and public health consequences.

## Data Availability

The datasets and scripts used in this study are available from the corresponding author upon reasonable request.
